# The Effect of Solvent Treatment on the Performance of Various Carriers in Dry Powder Inhalations Containing Salbutamol Sulphate

**Published:** 2013-07

**Authors:** Mohammad Reza Siahai Shadbad, Leonie Millen, MN Momin, Ali Nokhodchi

**Affiliations:** 1 Drug Applied Research Center and Faculty of Pharmacy, Tabriz University of Medical Sciences, Tabriz, Iran; 2 Medway School of Pharmacy, University of Kent, Chatham, ME4 4TB, Kent, UK

**Keywords:** Deposition test, Dry powder inhaler, Lactose, Mannitol, Solvent treated, Solid state characterizat- ion

## Abstract

***Objective(s)*** ***:*** It has been suggested that the efficiency of dry poder inhaler (DPI) is generally low. Therefore, the aim of the present research work was to use the solvent treatment of the carrier in DPIformulations to see the possibility of inducing desirable characteristics.

***Materials and Methods: ***Lactose sieve fractions of 63-90 µm were submerged in ethanol or 80% v/v ethanol, methanol or propanol. Lactose crystals were then blended with either 1% w/w or 4% w/w salbutamol sulphate using a Turbula mixer. Drug detachment was studied using a multistage liquid. Laser particle size analyzer, DSC, and pycnometer were used to characterize the treated lactose and mannitol samples. SEM was used to study surface morphologies. In case of mannitol as a carrier only ethanol was used as a solvent.

***Results: ***SEM images displayed less rugosities and increased surface smoothness after submersion. Although the tomahawk like shape remained fairly constant in most lactose samples, the solvent treatment changed the shape of mannitol particles which was proved by SEM results. Deposition results showed that the type of solvent had an influence on fine particle fraction. In addition, the payload had also a big impact on fine particle fraction values. Generally, treated samples showed better performance compared to untreated samples. Solid state analysis by DSC showed that no major changes occurred in the treated samples compared to untreated samples.

***Conclusion: ***The solvent treated method can be used as an approach to improve the performance of carriers such as lactose and mannitol in dry powder inhaler.

## Introduction

Traditional delivery systems for salbutamol sulphate, primarily was the metered-dose inhaler (MDI), but due to environmental concern regarding the use of chlorofluorocarbon (CFC) propellants other devices such as CFC free and dry powder inhalers (DPI) have since been developed (1).

It has been suggested that the efficiency of DPI’s is questionable. In some cases only 10% of the inhaled dose has been reported to reach the alveoli. The deposition of the dose is dependent on two major factors, the patient (i.e. technique & anatomical properties of the respiratory tract) and the physical properties of the aerosol cloud, (i.e. the formulation and device) (2). Formulation of the powder is equally if not more important than the design of the device. 

A typical dry powder formulation consists of a drug and carrier. The carrier is usually alpha lactose monohydrate (3). The carrier is used to improve the flow of drug. The drug to carrier ratio is usually 1:67.5 (w/w) (4). The carrier fraction used generally lies between 60-90 µm and the drug 1-5 µm (5). After inhalation the carrier should ideally stay in the device or be deposited in the oropharyngeal region due to its size, allowing the drug to efficiently detach and continue to the lower fraction of the lungs. Therefore, drug detachment from the carrier is an imperative part of drug delivery of DPI’s and is a focus of much research today. Interactions exist between the drug and carrier which are predominantly Van der Waals, but electrostatic and capillary are also present (6). 

The strength of these forces is dependent on physiochemical properties of the particles such as particle size, shape, surface morphology, hygroscopicity, and contact area (4). Such forces must be overcome on inhalation to ensure drug detachment from carrier and deposition in the lower part of the lungs. 

It had been suggested that a way to improve the bioavailability and dispersion of the drug is to use carriers with desirable physiological and physioche-mical characteristics. One such method is the recrystallisation of carrier particularly lactose (7-8) and mannitol (9-11). 

The aim of this study is to investigate the effects of solvent treatment of the carrier on the aerosolization performance of dry powder inhalers containing salbutamole sulphate. In light of the above aim, in the present study the effect of different types of alcohol with different strengths on the properties and morphology of lactose and mannitol was investigated. 

## Materials and Methods

***Materials***

Salbutamol sulphate (Ivax, UK), mannitol (Roquette, France), and lactose monohydrate (DMV international, UK) were used. Ethanol, isopropyl alcohol and methanol were purchased from Fisher scientific, UK. 

***Preparation of carrier***

Carrier fractions 63-90 µm were prepared by 10 minutes of mechnaical sieving. Half of the lactose sieve fraction was treated with aqueous ethanol solution, 96% v/v and the other with 80% v/v ethanol, to remove impurities and adhering fines from the particles and reduce the surface irregularities in order to produce carrier crystals with increased surface smoothness. This process was then repeated with methanol and propanol. For this treatment 100 g of lactohale particles (lactose monohydrate) was added to 1 litre of one of the alcohol solution and the mixture stirred for 10 min and then filtered (pore size 0.45 µm). The filtered residue was dried for 16 hr at 40^o^C on a tray in the oven and the dried fraction was passed through a 90 µm sieve to remove agglomerated lactose particles. Two duplicate batches were prepared for the next step. All experiments were carried out at a temperature of 25^o^C and a relative humidity of 50%. A control was also prepared which was not treated with any alcohol.

Mannitol was also treated in identical manner, but only ethanol was used as a solvent. 

***Blending of lactose with Salbutamol***

Each of the lactose sieve fractions was mixed with either 1.0% or 4.0% (w/w) salbutamol sulphate in a stainless steel container of 160 ml, using a turbula mixer (TZF 06034, Willy A Bachofen AG Switzerland) at 90 rpm for 10 minutes. The batch size was 25 g. 

***Characterisation of mixture***

Surface morphologies were studied using an electron scanning microscope (SEM) (Stereoscan 360– Cambridge instruments UK LTD, Edward sputter coater S150B)*. *Powder particles were scattered onto aluminium stubs with double sided adhesive carbon , thinly coated in gold for 2 minutes at 1kv and 30 milliamps then entered into the scanning electronic microscope. An image was then generated at different magnifications.

***Density and powder flow measurements***

The true density (ρ_true_) of all lactose samples was measured using an ultrapycnometer 1000 (Quantachrom, USA) using helium gas at an input gas pressure of 19 psi and an equilibrium time of 1 min. Each sample was weighed and an average of 3 experiments was conducted per sample at 5 pulses per minute. Therefore, the results are the mean and standard deviation of 3 runs.

Carr’s index (CI) was measured for all lactose powders as an indication of powder flowability (12, 13). 

Briefly, each powder was filled into a 5 mL measuring cylinder and after recording the volume (bulk volume) the cylinder was tapped 100 times and the new volume was recorded (tap volume). A preliminary experiment showed that 100 taps was sufficient to attain the maximum reduction in the volume of powder bed. The bulk density (ρ_b_), tap density (ρ_t_), and Carr’s Index (CI, Eq. 4) were measured: 

$\mathrm{CI}=(\frac{{\rho_{t}}_{-}\rho_{b}}{\rho_{b}})\times 100$                      Eq. 1

***Particle size distribution***

Particle size distribution of starting materials and blends were measured with a Sympatec HELOS compact KA laser diffraction apparatus (Sympatec, Clausthal-Zellerfeld, Germany), using a RODOS dry powder dispenser (at 2 bars). 

The samples were measured for accumulative distribution as well as a number of parameters including volume mean diameter (VMD) and span value (calculated as D_90%_ - D_10%_/ D_50%_). Each individual result was quantified and tabulated for percent frequency and cumulative frequency.

***Differential scanning calorimetry (DSC)***

DSC experiments were conducted using a Differential Scanning Calorimeter (DSC 822e Mettler Toledo Inc.)*.* Aluminium hermetic DSC pans were used throughout the study. The mass of the empty pan was weighed with sample to ensure the total did not exceed a weight of 4- 8 mg, they were then sealed with a toggle press at 10 N and punctured to release air. After sealing the pans were placed in the DSC furnace. 

Before each measurement the sample was allowed to equilibrate for 5 minutes at 25^°^C and was then heated to 250^°^C for lactose and 350^°^C for salbutamol at a heating rate of 10^°^C. min^-1^ under nitrogen gas. The instrument was calibrated with indium and zinc. 

**Table 1:** Micromeritics properties of lactose and mannitol samples treated with different solvents

Sample	True density (g/cm^3^)	VMD(µm)	Span	Carr’s Index (%)	Flow behaviour
Lactose only	1.543±0.011	74.39	1.18	5.0	Excellent
Treated with ethanol	1.547±0.013	86.02	1.00	17.5	Fair
Treated with 80% ethanol	1.547±0.010	69.66	1.28	20.0	Fair
Treated with methanol	1.551±0.013	75.88	1.16	17.5	Fair
Treated with 80% methanol	1.537±0.014	75.04	1.23	17.5	Fair
Treated with propanol	1.464±0.010	72.09	1.21	17.3	Fair
Treated with 80% propanol	1.437±0.008	69.40	1.21	17.5	Fair
Mannitol only	1.576±0.016	32.36	2.40	5.1	Excellent
Treated with ethanol	1.577±0.012	29.90	2.50	2.5	Excellent
Treaterd with 80% ethanol	1.601±0.009	27.19	2.48	5.0	Excellent

**Deposition studies**

Powder pulmonary deposition profiles of all dry powders were assessed *in vitro* using a Multi Stage Liquid Impinger (MSLI) equipped with a USP induction port (Copley Scientific, Nottingham, UK). Before operating, 20 mL of distilled water was introduced to MSLI stages 2, 3, 4 and 5 in order to make the collection surfaces wet. A filter paper (Whatman®; pore size <0.45 µm) was introduced in stage 5 of the impinger. The dose was entered into a standard inhaler (Airmax), which has been fitted to a moulded rubber mouth piece and attached to the throat piece of the impinger. Once the assembly had been checked and found to be airtight and vertical, the vacuum pump was switched on. The pump was allowed to-run for 4 seconds in which the dose was released. A total of 50 actuations were taken per sample, (according to pharmacopoeia guidelines).

The inhaler containing the formulation blends was fitted in a mouthpiece adaptor and then attached to the impinger induction port (IP). A pressure drop of 4 kPa was found to be induced through the impinger by operating at a flow rate of 60 L/min. The time interval over which actuation occurred was about 4 s, as calculated using equation 4:

Time = 240/ flow rate 

After 50 actuations for each sample, the water present on each stage was collected and then each stage was washed further with more distilled water several times (the final volume of water used to wash each stage was 100 mL). The powder deposited on the induction port was also collected by washing with water and the final solution adjusted to 100 mL. Each inhaler with its mouthpiece adapter was also washed thoroughly and the final washing solutions also constituted to 100 mL. The amount of salbutamol sulphate deposited on the inhaler and mouthpiece adaptor, induction port and each individual impaction stage of the MSLI was then quantified by UV. 

FPF was calculated from that plot as the cumulative amounts of drug with an aerodynamic diameter ≤ 5 µm taken as a percentage of the emitted dose. 

## Results

***Micromeritics behaviour of lactose and mannitol powders***

The true density of all lactose and mannitol samples (Table 1) showed that the solvents used to treat the samples had no remarkable effect on true density of powders. The effects of solvent treatment on the flow behaviour of lactose and mannitol samples were listed in Table 1. Carr’s index is a simple index that can be determined on small quantities of powder and can interpret the flow properties of the sample (12, 13). Good flow property for carriers is important in inhalation to make sure that the formulations containing drug particles can flow into capsule or inhaler and also from the inhaler to the mouth. As flow is greatly affected by factors such as size, shape, density and surface roughness, it is expected that the solvent treatment should have an important effect on the flowability of powders. All mannitol powders displayed excellent flow, whereas, the treated lactose samples lost their excellent flow properties and was reduced to fair flow. 

**Figure 1:**
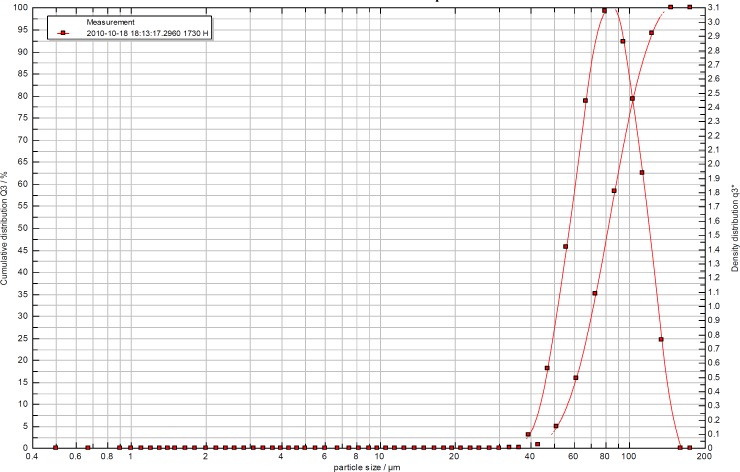
Particel size distribution of lactsoe powder

Table 1 shows that, generally, lactose or mannitol powders treated with 80% of the solvent showed smaller VMD. This could be due to the higher solubility of sugars in the presence of water (solubility of mannitol increased from 19.15 to 385.4 mg/Lit when the amount of ethanol decreased from 100 to 80%) which make the particles smaller. Similar reduction in the solubility was reported in case of lactose (14). For a dry powder inhalation a VMD (volume mean diameter) of between 70 and approx 120 µm is advisable (4). Most samples fell within this range apart from all mannitol samples which were either extremely lower than this. All powders showed similar pattern in terms of particle size distribution and an example was shown in Figure 1 for untreated lactose sample.

***Scanning electron microscope (SEM)***

**Figure 2:**
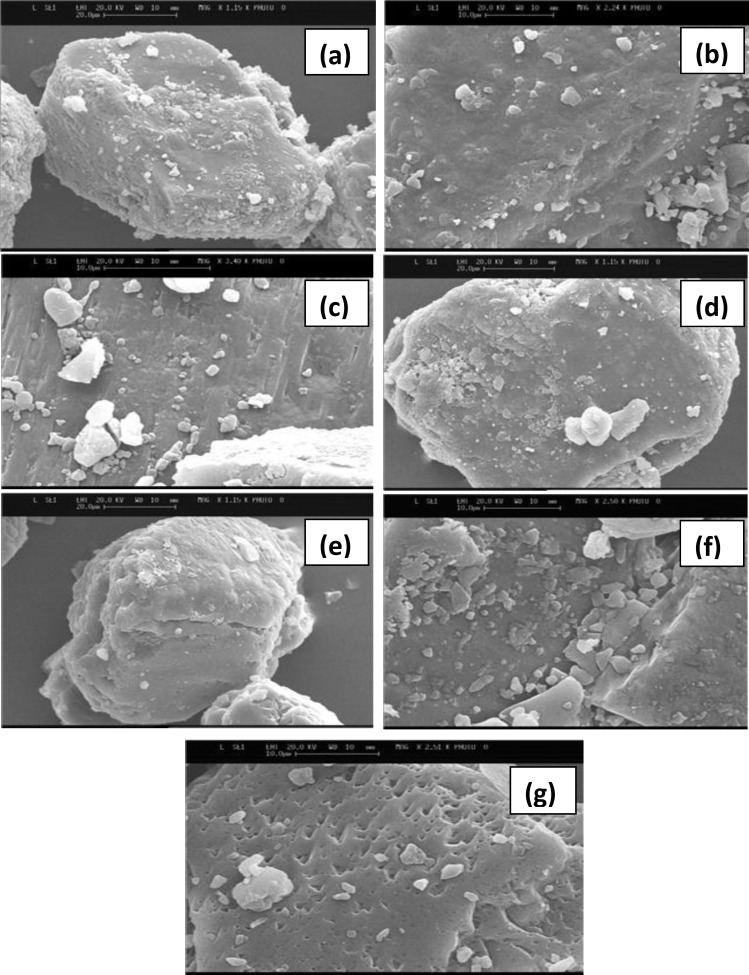
SEM images of various lactose samples treated with various organic solvents; (a) untreated lactose; (b) treated with ethanol; (c) treated with 80% ethanol; (d) treated with methanol; (e) treated with 80% methanol; (f) treated with propanol; (g) treated with 80% propanol

**Figure 3:**
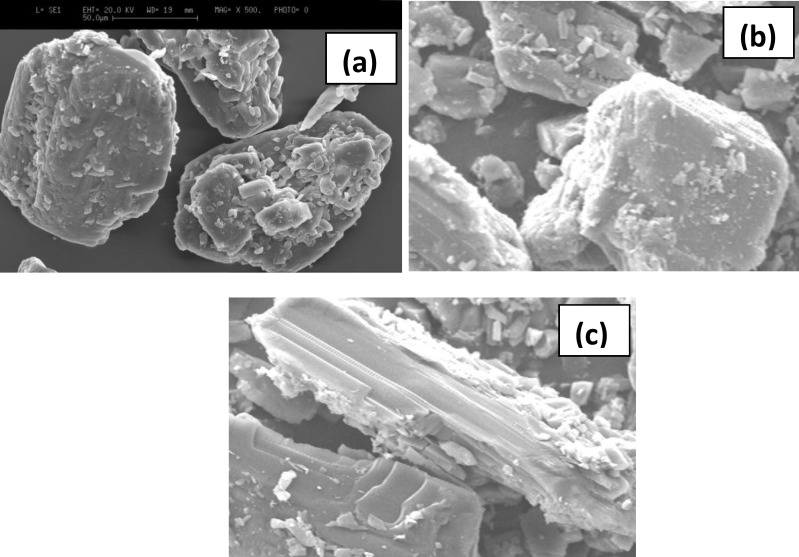
SEM images of mannitol treated with ethanol; . (a) untreated mannitol; (b) treated with ethanol; (c) treated with 80% ethanol

Images of all lactose and mannitol powders were shown in Figures 2 and 3, respectively. An appearance of increased smoothness in some of the samples is evident. A tomahawk shape was displayed by most particles which remained fairly consistent before and after submersion. Ethanol treated sample (Figure 2b) and 80% v/v treated lactose sample (Figure 2C) produced similar surfaces. When considering the methanol treated lactose, both 80% v/v (Figure 2e) and methanol (Figure 2d) tended to be very regular in size with smoother surfaces compared to untreated lactose. Propanol 80% v/v (Figure 2g) treated lactose also displayed porous surfaces and this was not observed for the samples treated with pure propanol (Figure 2f). 

In the case of mannitol, SEM results showed that the solvent had changed the morphology of the mannitol particles (Figure 3) and more elongated particles were obtained when it is treated with 80% ethanol.

**DSC studies**

**Figure 4:**
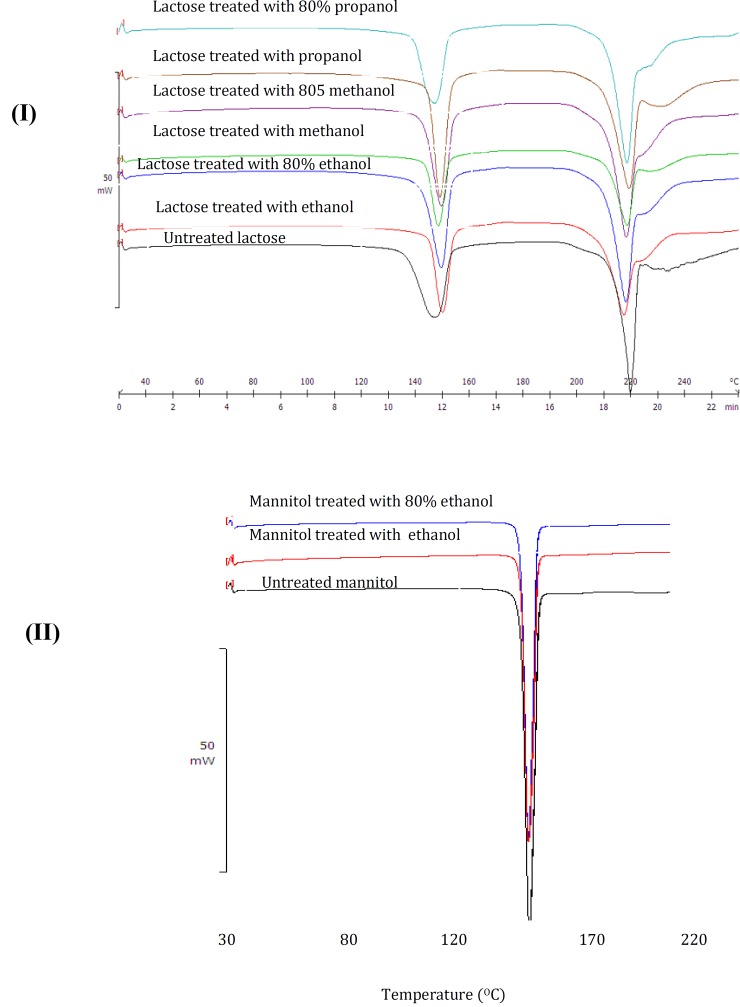
DSC traces of lactose and mannitol samples

Untreated lactose displayed two peaks (Figure 4I). The first peak at around 140 ˚C corresponds to the elimination of water (5). The second peak at 218 ˚C demonstrates the melting point of lactose. Most lactose blends also displayed an exothermic peak at approximately at approximately 170 ˚C, displaying the unstable nature of the lactose crystals (5).

Comparison between the DSC thermogram of mannitol before and after the submersion revealed that the position of the peaks was not significantly changed (Figure 4II). Mannitol displayed no changes in its melting points for all the samples. The peak at 166.90 ˚C corresponds to its melting point (Figure 4II). The results showed that all treated mannitol showed similar peak as untreated mannitol.

***Deposition studies***

The effect of the type of solvent on the aerosolization behaviour of formulation blends in terms of fine particle fraction (FPF) is shown in Table 2. The table also shows the effect of payload on FPF for all treated and untreated samples. The results showed that the type of solvent and also payload has significant effect (*P* < 0.05) on FPF. 

## Discussion

Dry powder formulations for inhalation often consist of micronised drug (1–5 µm) and inert coarse carrier particles (63-90 µm). The carrier particles are used to aid the flow and dispersion of the highly cohesive drug particles (4). Interaction forces exist between drug and carrier such as Van der Walls (6) that must be overcome on inhalation to ensure the drug is sufficiently detached from the carrier and effectively delivered to the lower respiratory tract. Research to date suggests that by gaining control over the properties of the carrier, much control can be gained over these interaction forces. Researchers have thought to control these properties by influencing the carrier payload (14), mixing time (15), bulk properties (2), and the surface properties (7, 9, 15, 16). 

**Table 2:** Micromeritics properties of lactose and mannitol samples treated with different solvents

Sample	Fine particle fraction (%)	
	1% pay load	4% pay load
Lactose only	34.2 48.1 45.9 50.1 59.3 44.5 42.1 52.0 56.5 45.7	44.1 53.1 48.5 71.5 52.9 52.4 70.9 53.8 54.8 55.8
Treated with ethanol		
Treated with 80% ethanol		
Treated with methanol		
Treated with 80% methanol		
Treated with propanol		
Treated with 80% propanol		
Mannitol only		
Treated with ethanol		
Treaterd with 80% ethanol		

The present research aimed to investigate how treating the surface of the carrier to remove impurities by the solvents can create a smoother surface that will aid in reducing these drug-carrier forces. Three different solvents (methanol, ethanol and propanol) either in pure or 80% strength (20% water) were used for lactose carrier submersion. Two drug strengths of salbutamol sulphate (1% w/w and 4% w/w) were also used. Results were compared to that of an alternative carrier mannitol, where only ethanol was used as a solvent, to ascertain whether mannitol is an effective alternative as a carrier.

Overall flow properties of all blends were not compromised after treatment with the solvents. Although, flow was reduced for the carriers from excellent to fair after submersion which could be due to the adherence of lactose particles to each other as a result of drying after washing. This was not the case for mannitol samples as the excellent flow was regained after blending salbutamol sulphate (SS) to the carrier (data was not shown).

The densities of the carriers either remained the same or were slightly increased after submersion. But the slight increase in the true density was not significant (*P*>0.05). Generally, high densities equate to better flow. However it can be said that the overall removal of impurities (in the case of lactose they are aldehydes, reducing sugars, proteins and some fats, in the case of mannitol they are polypeptide degradation impurity, oxidizing agents and reducing sugars) after submersion should reduce the density. This was only displayed by propanol treated blends, which may be attributed to the removal of impurities or be due to the porous Volume mean diameter’s (VMD) decreased after structure that came as a result of submersion (Figure 2g). 

Volume mean diameter^,^s (VMD) decreased after treatment with the solvents containing 20% water and 80% organic solvent. A slight reduction in particle size for 80% solvent could be due to the presence of water in this solvent which could make the particles smaller as a result of dissolution of the carrier surfaces. 

SEM images showed that after submersion the overall appearance did appear smoother and the ‘tomahawk’ like shape remained constant. This was consistent with Dickhoff *et al* (15) who claimed that submersion neither changes the size or the shape of particles, and Zeng *et al *(16) who found a smoother shiny appearance of lactose after recrystallization with carbopol gels. There was also a presence of some fines on lactose particles after submersion (Figure 2), which was also evident on the particle size distribution experiments, where increasing numbers of particles fell below the size of the sieve mesh used (63-90 µm), after submersion (Table 1). Steckel and Muller (18) suggested that by varying the particle size or drug content the fine particle fraction can be changed. 

Unlike lactose, mannitol blends did display a change of shape, from irregular or cube shaped (Figure 3b) to more elongated shape (Figure 3c) which was evident from the SEM images. It has been shown that, generally, needle shaped carrier increased the fine particle fraction of formulation blends containing mannitol-salbutamol sulphate (10). This contradicts the results obtained in the present study. In the present study, mannitol treated with 80% ethanol showed rough surfaces compared to untreated mannitol, hence low fine particle fraction. In the study carried out by Kaialy *et al* (10) the main reason for elongated mannitol showing higher FPF was smooth surfaces. This suggests that if the elongated mannitol obtained in the present study had smoother surfaces it would have shown higher FPF compared to other mannitol particles. 

Overall, the present results showed that the treated samples performed better than untreated samples in DPI formulations. This was in good agreement with the data provided by Iida et al. (19) and Zeng *et al* (16). This then disproves Dickhoff *et al *(6) who found that the carrier produced was almost too smooth and decreased detachment. The submersion treatment of the carrier under defined conditions is favorable for drug particle detachment from the carrier. From the data reported in Table 2, it is very difficult to say why, for example, propanol should act better than others as there are so many factors that can change the performance of carrier in DPI formulations such as the amount of fines (20), the smoothness of the carrier surfaces and the surface rugosity (21), the size of carrier (22), flowability of carrier (10), and reduction of binding sites (23).

**Figure 5:**
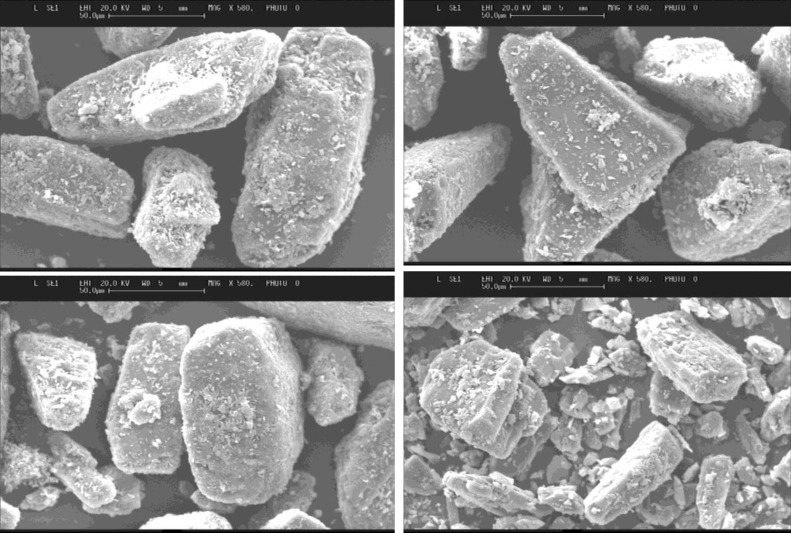
SEM images of formulation blends of treated carrier with 4% slabutamol sulphate; (a) lactose tretaed with ethanol; (b) lactose tretaed with methanol; (c) lactsoe tretaed with propanol; (d) mannitol tretaed with ethanol

At higher drug payloads (4% w/w) blends produced a more significant FPF than 1% blends, which is consistent with Dickhoff *et al* (15) who found decrease in carrier residue (the amount of residual drug on the carrier after inhalation) with increasing payload, this therefore equates to better detachment. Dickhoff *et al* (15) and Young *et al *(24) described that the increase in FPF was as a result of an excess of drug particles relative to the number of bonding active sites, this reduces the mean adhesive force in the mixture between drug and carrier particles. SEM images of formulation blends containing 4% salbutamol showed that the drug particles are distributed very well on the carrier surface which is evident from Figure 5.

Table 2 also showed that the overall performance of mannitol was found to be as good as lactose articularly at high payload) regardless of the treated mannitol. This is consistent with the published results suggesting manitol has a great potential as an alternative sugar to replace lactose in inhalation formulations (9, 18, 25).

## Conclusion

The results suggest that submersion treatment of the carrier under defined conditions, such as the choice or strength of the solvent or drug payload, is favorable for drug particle detachment from the carrier. This is due to the removal of rugosities and making carrier surfaces smoother. The present study also suggests that mannitol can be an alternative for lactose carrier in dry powder inhalation formulations. In conclusion, the efficiency and reproducibility of drug delivery by dry powder inhalers can be improved using carrier particles of precisely defined morphological features.
